# Factors Affecting the Propensity of Tsetse Flies to Enter Houses and Attack Humans Inside: Increased Risk of Sleeping Sickness in Warmer Climates

**DOI:** 10.1371/journal.pntd.0002193

**Published:** 2013-04-25

**Authors:** Glyn A. Vale, John W. Hargrove, Andrew Chamisa, David R. Hall, Clement Mangwiro, Stephen J. Torr

**Affiliations:** 1 Natural Resources Institute, University of Greenwich, Chatham, United Kingdom; 2 Southern African Centre for Epidemiological Modelling and Analysis, University of Stellenbosch, Stellenbosch, South Africa; 3 Division of Tsetse Control, Harare, Zimbabwe; 4 Bindura University of Science Education, Bindura, Zimbabwe; 5 Vector Biology Department, Liverpool School of Tropical Medicine, Liverpool, United Kingdom; 6 Warwick Medical School, University of Warwick, Coventry, United Kingdom; IRD/CIRDES, France

## Abstract

**Background:**

Sleeping sickness, or human African trypanosomiasis, is caused by two species of *Trypanosoma brucei* that are transmitted to humans by tsetse flies (*Glossina* spp.) when these insects take a bloodmeal. It is commonly assumed that humans must enter the normal woodland habitat of the flies to become infected, but recent studies found that tsetse frequently attack humans inside buildings. Factors affecting human/tsetse contact in buildings need identification.

**Methodology/Principal Findings:**

In Zimbabwe, tsetse were allowed access to a house via an open door. Those in the house at sunset, and those alighting on humans in the house during the day, were caught using hand-nets. Total catches were unaffected by: (i) the presence of humans in the house and at the door, (ii) wood smoke from a fire inside the house or just outside, (iii) open windows, and (iv) chemicals simulating the odor of cattle or of humans. Catches increased about 10-fold with rising ambient temperatures, and during the hottest months the proportion of the total catch that was taken from the humans increased from 5% to 13%. Of the tsetse caught from humans, 62% consisted of female *G. morsitans morstans* and both sexes of *G. pallidipes*, *i.e.*, the group of tsetse that normally alight little on humans. Some of the tsetse caught were old enough to be effective vectors.

**Conclusion/Significance:**

Present results confirm previous suggestions that buildings provide a distinctive and important venue for transmission of sleeping sickness, especially since the normal repellence of humans and smoke seems poorly effective in such places. The importance of the venue would be increased in warmer climates.

## Author Summary

To identify factors affecting the contact between tsetse and humans in buildings, we caught tsetse that (i) accumulated in a large thatched house in Zimbabwe, and (ii) alighted on humans in the house during the day. In accord with earlier work, the numbers accumulating increased about 10-fold with rising ambient temperature. However, it was surprising that the numbers were unaffected by the presence of humans or artificial human odor in the house, or by wood smoke or a simulation of ox odor, since these factors can affect greatly the catches at baits in woodland. Tsetse that alighted on humans in the house contained a high proportion of those classes of tsetse that seldom alight on humans. Some of the alighting flies were old enough to be vectors of sleeping sickness. Our results emphasize that buildings are venues for important and distinctive contact between humans and tsetse, and that the risk of disease transmission there may be greater in warmer climates.

## Introduction

Tsetse flies (*Glossina* spp.) feed exclusively on vertebrate blood, and in so doing they can transmit species of trypanosome (*Trypanosoma* spp.) that cause the diseases of nagana in domestic animals and sleeping sickness in humans [1]. The latter disease, also known as human African trypanosomiasis (HAT), is caused by two subspecies of *T. brucei*: *T. b. gambiense* and *T. b. rhodesiense*. Between them, these two parasites account for several thousand new recorded cases of HAT each year, but since diagnosis and reporting are often poor it is likely that the true number of cases is much greater [2].

While it is common to assume tacitly that almost all contact between humans and tsetse occurs when humans enter the woodland habitat of the flies, two recent papers [3], [4] showed that much contact occurs in Zimbabwe when tsetse flies, *G. morsitans morsitans* and *G. pallidipes*, approach or enter buildings in large clearings. Moreover, these papers indicated also that a high proportion of the tsetse attacking men inside buildings were females, *i.e.*, the sex that usually forms a very small proportion of the tsetse caught on humans in woodland. In consequence, it seems that the contact between tsetse and humans in houses and other buildings is an important and distinctive venue for the transmission of sleeping sickness. Hence, we need to know what factors affect the propensity of tsetse to enter buildings, and whether we can reduce the human/fly contact inside.

First attempts to answer the above questions [4] suggested that at all times of year some of the tsetse responding to various types of house did so in a phase of behavior analogous to the response to host-like traps; other flies entered the houses to find a cool refuge from high temperatures during hot weather. This preliminary work was performed with houses that were occupied for only a few minutes every two hours, and so was useful in showing that the houses were themselves attractive, irrespective of a prolonged human presence. However, it needs to be shown to what extent the more permanent presence of humans in houses affects the responsiveness of the flies. For example, given that humans produce an odor that can reduce markedly the catches from hosts and host-like objects [5], it might be expected that human odor would decease substantially the numbers of tsetse entering houses. Moreover, since wood smoke reduces the catch of tsetse from traps to virtually nil [6], the smoke from domestic fires might drastically inhibit house entry. Against this, the contamination of the house or human clothing with residual odor that originates from domestic animals and which is known to be effective with baits in woodland [7] might be expected to increase the entry.

Present work elucidated the impact of human presence, smoke and odor attractants on the magnitude and composition of samples of *G. m. morsitans* and *G. pallidipes* caught in a house at various seasons and studied the extent to which the flies inside were responsive to humans.

## General Methods

### Ethics

All work was performed at Rekomitjie Research Station in the Mana Pools National Park of the Zambezi Valley of Zimbabwe. In the last 54 years no case of HAT has been recorded as contracted at the station, despite the good diagnostic facilities there. Hence, the station offers the opportunity to study those aspects of tsetse behavior which could be expected to be associated with HAT transmission elsewhere, but without the Rekomitjie personnel being subjected to a material risk of infection. All persons used as catchers or baits in the experiments were permanent pensionable employees of the Division of Tsetse Control, Government of Zimbabwe, and were given regular updates on the purpose and results of the studies. Before recruitment, the Division explains the nature of the work, the risks associated with tsetse, other disease vectors and wild animals, and warns of the social hardships attending life on a remote field station. Recruits sign a document indicating their informed consent to perform the work required. This document is held by the Division. All experiments were given ethical approval by the Division's Review Committee for Rekomitjie.

### House

Studies were performed in a thatched white-painted house, 7.5 m wide and 19.5 m long, in the bush-cleared grounds of the station. Details of the station, the floor plan of the house and the diurnal variations of temperature in the house, are given in [4]. For present purposes it need be noted only that the house had a net-windowed veranda along the whole of its West side, *i.e.*, the predominantly downwind side, in the middle of which was a door opening to the outside; on all other sides of the house there were glazed windows. At night the door and windows were closed. Unless stated otherwise, the door was always open during the day, *i.e.*, from sunrise to sunset, and the windows and a second door on the East side were shut day and night. Under such circumstances the West door was the only apparent point of tsetse entry. All internal doors were always open.

### Treatments

Sometimes the house was empty and at other times occupied for the whole day by a team consisting of three adult Africans, usually one male and two females. Each team worked two alternating shifts of about 3 hrs each. At the change of shifts, the newly arriving people stopped just outside at the door, used hand-nets to catch any tsetse that had come with them, killed and discarded such flies and then entered the house to replace the previous team. The individual humans comprising each team varied from day to day, depending on which persons were available, so that the whole study used five male and nine female individuals. No separate records were made of the catches from individual humans since tsetse often flitted between the persons before being captured. For much of the time the people sat on chairs on the veranda, 3–5 m from the door, so that their odor occurred at or near the door. The following treatments were sometimes used in the presence or absence of humans in the house.

1. Artificial ox odor, called AOP and consisting of 100 mg/h of acetone, 1 mg/h of 4-methyl phenol, 0.5 mg/h of 1-octen-3-ol and 0.1 mg/h of 3-*n*-propyl phenol, was dispensed as described in [8]. The dispensers were placed on the doorstep, corresponding roughly with the fact that when used with traps they are normally located 50 cm downwind of the trap's entrance [6].
2. Artificial human odor, called AHO and involving 0.2 mg/h of geranyl acetone and 2 mg/h of 6-methyl-5-heptan-2-one, was released from individual sachets [6] placed in an open 210 ml glass beaker on the doorstep.
3. Smoke was produced from a smoldering fire of *Colophospermum mopane* logs, about 5 cm in diameter and 25 cm long, placed on a rusted steel tray 45 cm in diameter. In one experiment the fire was outside, just below the doorstep, and in another experiment it was inside the house, on the veranda, 1 m from the door. If humans were stationed in the house, the fire inside was kept going by these people. Otherwise the fire was tended briefly every few hours by a man who caught and discarded any tsetse following him before he entered the house.
4. To ensure that tsetse could not pass into the house without encountering a human very closely, an African male was stationed all day on the doorstep. This doorman was additional to any team of three people inside the house.
5. To encourage the flow of human odor out of the house, the windows on the East, *i.e.*, upwind, side of the house were open all day.

If people were inside the house, tsetse that alighted on them at any time of day were caught using hand-nets, but no attempt was made then to catch any other tsetse seen, *e.g.*, on the walls, at the windows or on any doorman present. Just before sunset, when the inside of the house was still suitably illuminated, the door and any open windows were closed and all tsetse remaining in the house were caught, either using hand-nets or by disturbing the flies so that they flew to the windows where they could be picked off manually. These catches were called the “house” catches, to distinguish them from those made from people in house. The total daily catch was the sum of tsetse caught from the house at sunset, plus any taken from people inside during the day. Dry bulb temperatures were measured in a Stevenson screen near the centre of the station, about 150 m from the house.

### Age of tsetse

Female tsetse were dissected to determine their ovarian category (0–7), which offers an index of age – category 7 being the oldest [9]. Male age was gauged from wing fray class (1–6) – class 6 being the oldest [10].

### Statistics

A number of experiments employed randomized block designs in which 2–4 distinctive treatments were allocated to a separate day within a of block of adjacent or nearly adjacent days, with a total of 8–17 blocks per experiment. Often the daily catches of each individual sex and species of tsetse were nil or very low. Thus, since the compositions of catches from the various treatments did not seem to vary greatly, the daily catches of males and females of *G. m. morsitans* and *G. pallidipes* were pooled to give larger catches for statistical analysis. Such analysis involved transforming the catches to log(n+1), but the catches were detransformed for reporting. Chi-squared tests were performed for the homogeneity of the distributions of catches between various categories of sex, species or reproductive condition. In some cases certain categories were pooled to ensure expected values > = 5. The term “significant” implies P<0.05. The 95% confidence limits of the percent composition of samples were calculated using the BinomHigh and BinomLow add-in functions of Microsoft's Excel.

## Experiments and Results

### Effect of treatments on catch size

The total catches, *i.e.*, from the house and from any humans inside, made in the separate experiments were surprising in showing no clear or consistent effect of the various treatments (Table 1). In particular, analysis of variance indicated that the mean daily catches were not increased significantly by artificial ox odor (AOP), nor reduced by smoke or artificial human odor (AHO). Admittedly, the ANOVA of Experiment 5 indicated a heterogeneity between means that was just significant, at P = 0.04, due primarily to the relatively low catch from the house plus humans treatment (second row of Expt 5, Table 1). However, this was the only experiment showing significant heterogeneity between means, and given that seven experiments were performed, it was not particularly unexpected that one of them would involve an observed effect that was just significant. Hence, to get a seemingly more reliable indication of the effect of humans it is pertinent to combine the data for all experiments in which humans were present and absent, *i.e.*, Experiments 1–6. In the 95 replicates with humans, the mean daily catch was 6.2 (95% CL 5.3–7.2) without humans, as against 6.0 (5.1–7.1) in the same number of replicates with humans. Thus, it appears that even if there is indeed a real effect of humans in houses it is likely to be small when compared with the 50–90% reduction in catches when humans are present near traps [6] or cattle baits in woodland [5].

**Table 1:** Catches of tsetse from the house, in seven separate experiments with various treatments.

Experiment and treatment	*G. m. mors.*		*G. pallid.*		Total	Mean	95% CL
	M	F	M	F			
**Expt 1, Aug/Sep 2010, 15 replicates**							
Nil	13	47	113	435	608	30.4	15.9–57.2
Humans	30	67	160	465	722	40.5	21.4–75.9
**Expt 2, Oct/Nov 2010, 8 replicates,**							
Nil	4	20	33	131	188	15.9	7.0–34.6
Humans	16	31	17	81	145	14.0	7.2–26.4
AOP^1^	4	25	40	123	192	17.9	8.2–37.9
AOP^1^+humans	18	38	28	146	230	24.4	14.2–41.2
**Expt 3, Jan/Feb 2011, 8 replicates**							
Nil	2	1	12	30	45	4.9	2.7–8.4
Humans	2	4	16	23	45	5.1	3.1–7.9
Fire outside	1	2	15	29	47	4.8	2.3–9.1
Fire outside+humans	3	2	20	25	50	5.3	2.9–9.1
**Expt 4, Mar/Apr 2011, 8 replicates**							
Nil	2	2	22	40	66	6.4	3.2–12.1
Humans	0	1	9	24	34	3.4	1.4–7.0
Fire inside	2	1	7	27	37	3.1	1.0–7.2
Fire inside+humans	0	1	14	15	30	2.7	0.9–6.1
**Expt 5, May/Jun 2011, 8 replicates**							
Nil	0	2	14	39	55	6.5	4.8–8.8
Humans	0	5	11	32	48	3.4	1.0–8.6
Windows open	1	8	28	24	61	5.5	2.2–12.5
Windows open+humans	3	6	28	30	67	7.9	8.9–10.6
**Expt 6, Jul/Aug 2011, 8 replicates**							
Nil	0	0	4	19	23	2.6	1.5–4.1
Humans	4	4	9	11	28	2.2	0.5–5.9
Doorman	0	3	2	21	26	2.5	1.0–5.3
Doorman+humans	2	6	8	11	27	2.6	1.0–5.5
**Expt 7, Sep/Oct 2011, 17 replicates**							
Nil	7	16	44	195	262	13.3	9.1–19.4
AHO^2^	5	22	61	233	321	16.4	11.6–23.0

1 Artificial ox odor.

2 Artificial human odor.

Total catches of male (M) and female (F) *G. m. morsitans* and *G. pallidipes* in all daily replicates of each treatment in each experiment, the daily mean of the catch of both sexes and species combined, and the 95% confidence limits of the mean. All humans except the doorman were inside the house; windows were closed unless stated otherwise.

It was especially surprising that the presence of a man at the door did not reduce catches – given that tsetse had to pass right by him in order to enter the house, and so were well exposed to his visual and olfactory stimuli. In further emphasis of the fact that the man at the door seemed to have no material effect, it is pertinent to examine the catch composition in his presence and absence. Without the man the total catch in the house was eight *G. m. morstitans* and 43 *G. pallidipes*, as against figures of 11 and 42, respectively, in his presence. This result contrasts with the fact that human baits in woodland are associated with gross reductions in the proportions of *G. pallidipes* in catches [5], [6].

### Distribution between humans and house

For the total of 95 days in which humans were in the house during Experiments 1–6, the numbers of tsetse caught from the humans throughout the day were compared with the catches from the house itself at the end of the day – the latter catches indicating the number of tsetse that had been in the house for up to 12 hrs but had not been caught from people in that time. The results (Table 2) showed a significant departure from a 50∶50 distribution of catches between the house and the humans, with each sex and species of tsetse. However, the number from the humans relative to the number from the house varied greatly. For male *G. m. morsitans*, most flies were caught from the humans; for female *G. m. morsitans* more were caught from the house, and that trend was taken much further by male and female *G. pallidipes*. This pattern of catches accords with the indications of much other work, that the propensity to alight on humans is greater for *G. m. morsitans* than for *G. pallidipes*, and greater for males than for females [5]. Thus, once tsetse are were in the house it seemed that much of the normal aversion to humans applied.

**Table 2:** Catches from humans in the house and from the house itself, in 95 days during Experiments 1–6.

Source	*G. m. morsitans*		*G. pallidipes*	
	Males	Females	Males	Females
Humans	49	61	8	11
House	29	104	312	852
Percent from humans	62.8	37.0	2.5	1.3
95% CL^1^	51.1–73.5	29.6–44.8	1.1–4.9	0.6–2.3

1 Confidence limits for percent caught from humans.

Catches from the humans were made during the day. Catches from the house were made at the end of the day.

Nevertheless, the composition of the catch from the humans in the house was peculiar, being distinct from that of catches from any other sampling system in common use at Rekomitjie. For example, the 15% of *G. pallidipes* in the catches was much less that the 40–90% commonly expected from refuges and traps [3], [11], but somewhat more than the 1% usually found in hand-net catches from men in woodland [3]. Moreover, while the 55% of females in catches of *G. m. morsitans* from the men was compatible with the high percents of females in catches of this species from traps and refuges [3], [11], it was greater than the percent normally caught by hand-nets from mobile baits, and much greater than the 5–10% usually associated with hand-net catches from men in woodland [3]. Hence, in keeping with the indication, above, that humans in the house caused little or no reduction in the numbers of tsetse entering, the conditions inside the house seemed to counter some of the repellence of humans.

Overall, the present sample of 129 tsetse from humans in the house contained a total of 62% (95% CL 53–70%) of those tsetse, *i.e.*, female *G. m. morsitans* and both sexes of *G. pallidipes*, that normally alight very little on humans. This proportion is even higher than the already high proportions of 17–47% (pooled value = 43%, N = 257) found in samples taken throughout the year from humans in buildings in two previous studies at Rekomitjie [3], [4].

### Season and temperature

**Figure 1:**
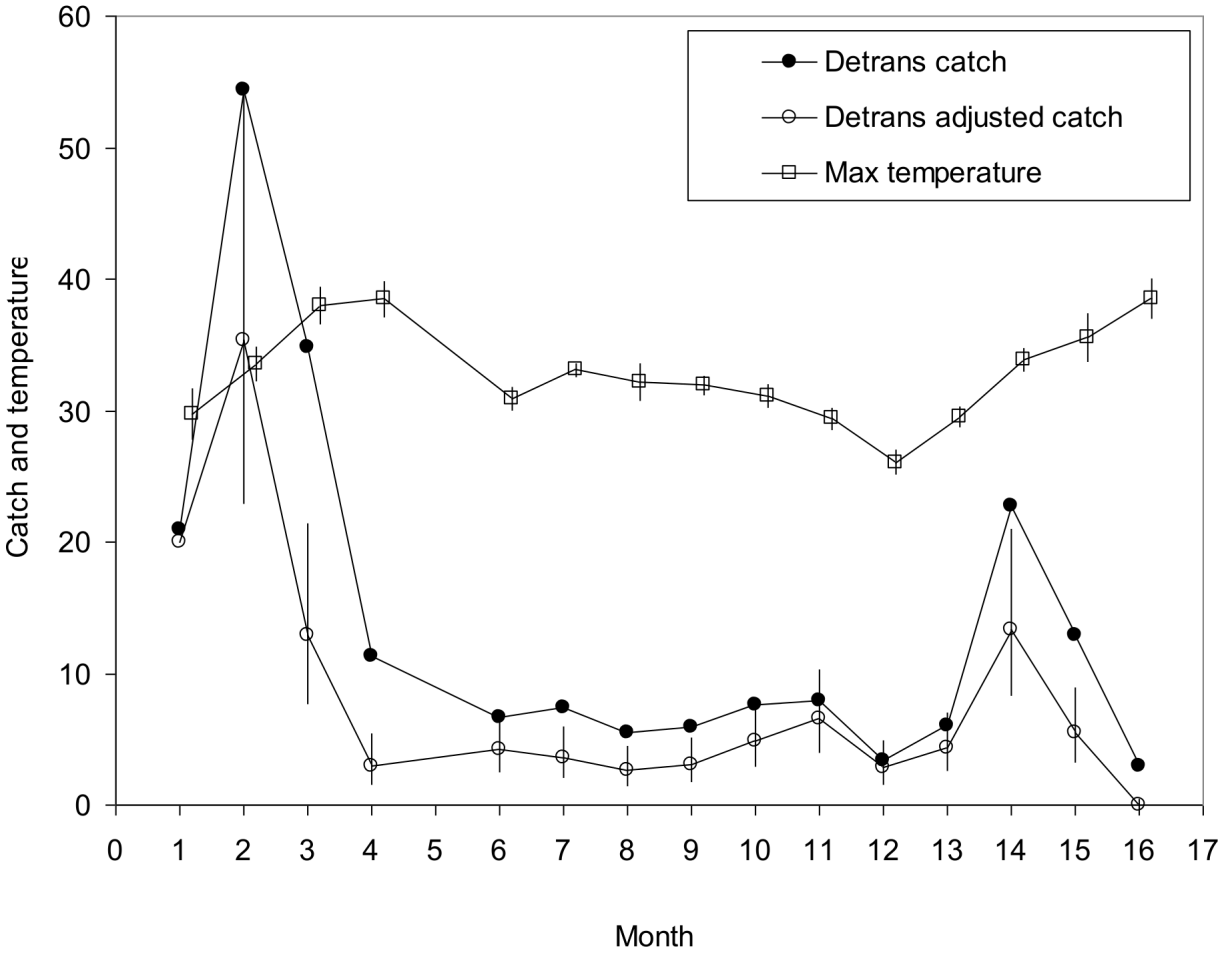
Monthly temperatures and catches from the house. Monthly data for detransformed mean daily catches of all tsetse from the house, mean maximum temperature, and detransformed catches adjusted for the effect of temperature. Bars through means indicate 95% confidence limits.

Given (Table 1) that the was no marked effect of the various treatments on the total catch from the house, *i.e.*, the catch from the house itself plus that from any people inside, the daily total catches of all treatments in each month were regarded as a single data set. This, together with extra data produced for the empty untreated house in November 2011, provided 12–18 (mean 16) daily catches within each month in the period August 2010 to November 2011, with the single exception that no data were available for December 2010. The detransformed mean daily catches (Fig. 1, Detransformed catch) showed that catches peaked in the early part of the hot season, *i.e.*, in September and October, consistent with the expectation from other work that catches would increase with temperature [4]. However, catches dropped sharply in November, despite high temperatures then, but according with the fact that tsetse densities vary throughout the year, with the greatest decline occurring in the late dry season [12].

A multivariate analysis of the transformed daily catches was performed to remove the effects of daily temperature and months. This showed a significant effect of daily temperature, such that when temperatures rose from the observed minimum value of 23.0°C to the greatest observed value of 42.5°C the catches increased 9.8 times. The effect of months, *i.e.*, the presumed effect of seasonal changes in tsetse densities, was shown by the monthly mean detransformed catches adjusted for temperatures within months (Fig. 1, Detransformed adjusted catch). As expected [12], there was a significant effect of months, with the apparent density of tsetse being greatest in September, and declining steeply during October and November, associated with the high mortality of tsetse during hot weather [13].

The monthly data for the numbers of tsetse caught from people in the house were less complete than the data set of Fig. 1, since humans were not deployed in the house in September to November 2011. Nevertheless, data were available for August 2010 to August 2011. These data indicated no marked seasonal change in the sex and species composition of the catches from humans. However, most flies were caught in the hot months of September to November, when the three-month total was 40 male *G. m. morsitans*, 49 female *G. m. morsitans* and 15 *G. pallidipes*, as against figures of only 9, 12 and 4, respectively in the other nine months, *i.e.*, August 2010 and January to August 2011. Looked at another way, the combined catch of all sexes and species of tsetse from the humans, as a percent of the combined catch from the house itself, was 12.8% (house catch = 815) in September to November, as against only 5.2% (482) in the other months, with the apparent seasonal effect on the distribution of catches between the humans and the house being significant.

### Diurnal patterns

**Figure 2:**
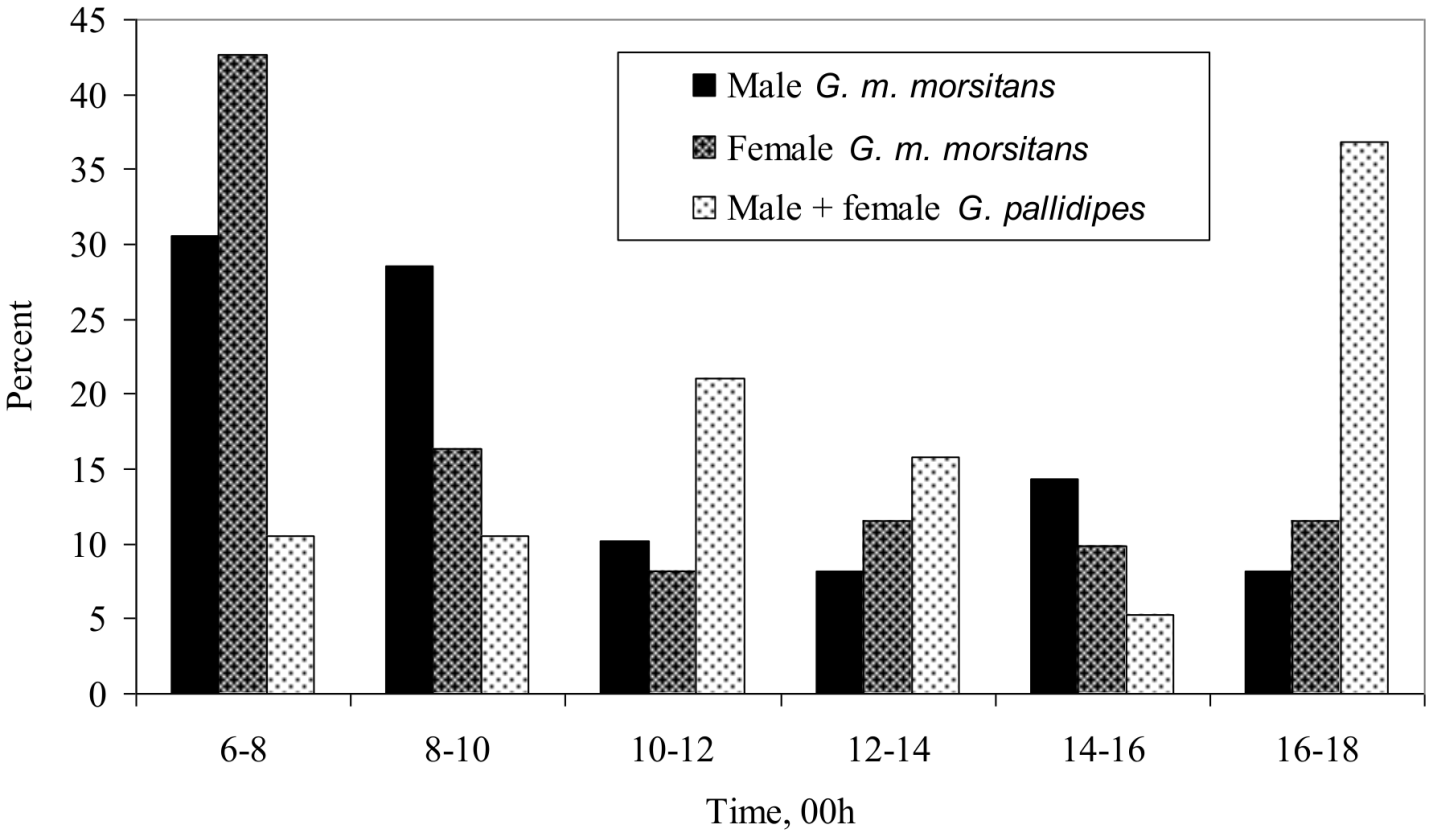
Diurnal distribution of catches of tsetse from humans in the house. Samples sizes of 49 for male *G. m. morsitans*, 61 for female *G. m. morsitans*, and 19 for male+female *G. pallidipes*.

All diurnal data relate to the catches from humans in the house – the catches from the house itself being made only at the very end of the day. Catches of *G. m. morsitans* from the people were greatest in the first four hours of the morning, and were roughly steady for the rest of the day, but for *G. pallidipes* the catches were concentrated in the evening (Fig. 2). At hosts or host-like traps in woodland, both species normally show a marked peak of availability in the evening [11], so that the absence of an evening peak with *G. m. morsitans* seemed a distinctive feature of the availability to humans in the house.

### Age of tsetse

**Figure 3:**
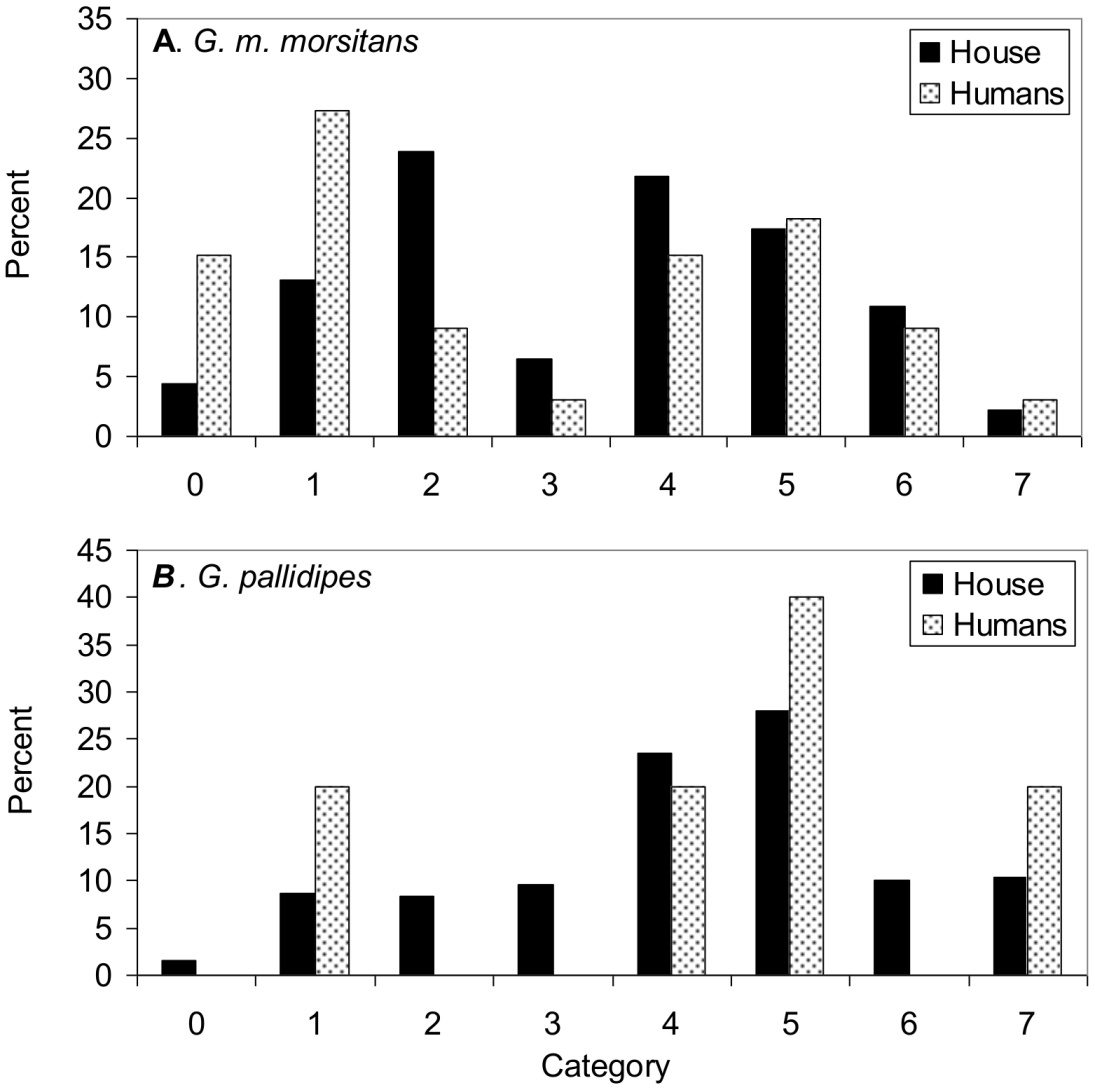
Distribution of ovarian categories in catches. Data for female *G. m. morsitans* (A) and *G. pallidipes* (B), taken from the house itself and from humans in the house. Sample sizes for *G. m. morsitans*: house 46 and humans 33; for *G. pallidipes*: 336 and 5, respectively.

The distribution of ovarian ages (Fig. 3) indicate that 42% (N = 33) of the female *G. m. morsitans* caught from humans were young, *i.e.*, in categories 0 or 1. This percent is significantly greater than the 17% (46) of young female *G. m. morsitans* from the house itself. Despite this, many of the female *G. m. morsitans* from the humans were in categories > = 4, suggesting that they were old enough to be potential vectors of HAT [14]. For *G. pallidipes*, the majority of the females were in the older categories, whether they were from people or the house, although the sample size (5) from the people was very small.

Wing fray classifications of males confirmed the indication that a relatively high proportion of the *G. pallidipes* from the house were old. Thus, 54% (N = 198) of the males of this species were in classes > = 3. This was significantly greater than the 31% (26) evident for male *G. m. morsitans*. Three male *G. pallidipes* were examined from humans, two flies being in class 1 and the other was in class 2, but this sample was too small to assess reliably the effect of age on the availability of male *G. pallidipes* to humans. However, of the 21 male *G. m. morsitans* examined from humans, 29% were in classes > = 3, suggesting that they were old enough to be potential vectors [14].

## Discussion

Buildings certainly do not provide the only points of contact between humans and tsetse, since that contact occurs also in woodland, especially when people travel on vehicles [3]. Nevertheless, our present results confirm previous suggestions that buildings can be important and distinctive venues for the transmission of HAT [3], [4]. The most distinctive feature confirmed was that the tsetse attacking people in houses contain high percents of those classes of tsetse, *i.e.*, female *G. m. morsitans* and both sexes of *G. pallidipes*, that usually alight relatively infrequently on humans in other venues. However, the percent of these tsetse on humans in the present work was particularly high, at 62%, being nearly half as great again as that found previously. Why is the percent so very high now? Present studies used many women as baits, whereas the previous work employed only men, but this is unlikely to be important since tsetse seem not to distinguish between men and women [5]. Perhaps, the more likely explanation is that in the present work the tsetse and humans were in each other's presence for up to 12 hrs, as against the few minutes in the earlier studies, so that the tsetse had more time to overcome their normal aversion to humans. Presumably, this involved an habituation to the repellence of humans, and/or a reduction in food reserves sufficient to make the flies less discriminating [3].

Two extra distinctions are now suggested. First, the numbers of *G. m. morsitans* available to humans in houses did not show the marked evening peak typical of the availability of tsetse to host-like baits in woodland. Although the sample size (110) involved was too small to indicate precisely the diurnal pattern of behavior, and the way it might have varied over the year, the result was still surprising since many tsetse would have accumulated in the house during the day, so that by evening the numbers potentially available to the humans would be relatively great. The intrigue is enhanced further by the fact that the evening peak was clearly evident with *G. pallidipes*. Second, there was the surprising and perhaps much more important fact that the numbers of tsetse caught in the house were not materially affected by the attractants and repellents that normally have a great impact on catches at baits in woodland. In particular, neither the humans in the house, nor the man at the door, nor the smoky fire inside or out, seemed to have any substantial effect on total catches from the house. Hence, it appears that entry into buildings is an especially determined response, firmly embedded in tsetse behavior. This raises the suspicion that the response is shown in a range of locations other than Rekomitjie, and is unlikely to be countered conveniently. For example, the use of insecticide-treated bed-nets is not likely to be effective since tsetse are inactive at night. It might be more beneficial to treat the inside of the house with insecticide, particularly the darker nooks where refuge-seeking tsetse concentrate [11], or to provide funnels on netted windows to permit tsetse to exit without letting them in.

Allowing that several peculiar, surprising and possibly important things have been found by the present limited studies with just one particular house, it might be expected that several more matters of consequence would be exposed by fuller studies conducted in a variety of buildings in different geographical locations, with a range of other tsetse species, and accompanied by studies of the nutritional status of flies doing different things in the houses. Such matters are currently under investigation in Zimbabwe and elsewhere. For the moment, however, it appears that ten times more tsetse can occupy buildings when temperatures rise, and that the responsiveness to humans among the flies in the building seems about two and a half times greater in hot weather. Thus, in warmer environments, including any that might be produced by climate change, the sleeping sickness risk associated with houses could be increased.
